# Comparison of a novel Compressed SENSE accelerated 3D modified relaxation-enhanced angiography without contrast and triggering with CE-MRA in imaging of the thoracic aorta

**DOI:** 10.1007/s10554-020-01979-2

**Published:** 2020-08-27

**Authors:** Lenhard Pennig, Anton Wagner, Kilian Weiss, Simon Lennartz, Michael Huntgeburth, Tilman Hickethier, David Maintz, Claas Philip Naehle, Alexander Christian Bunck, Jonas Doerner

**Affiliations:** 1 Institute for Diagnostic and Interventional Radiology, Faculty of Medicine and University Hospital Cologne, University of Cologne, Kerpener Straße 62, 50937 Cologne, Germany; 2 Philips GmbH, Hamburg, Germany; 3 Department of Radiology, Massachusetts General Hospital, Harvard Medical School, 55 Fruit St, White 270, Boston, MA 02114 USA; 4 Else Kröner Forschungskolleg Clonal Evolution in Cancer, University Hospital Cologne, Weyertal 115b, 50937 Cologne, Germany; 5 Adult Congenital Heart Disease (ACHD) Center, Clinic III for Internal Medicine, Department of Cardiology, Heart Center, Faculty of Medicine and University Hospital Cologne, University of Cologne, Kerpener Straße 62, 50937 Cologne, Germany

**Keywords:** Magnetic resonance angiography, Thoracic aorta, Non-contrast-enhanced magnetic resonance angiography, Connective tissue diseases

## Abstract

To compare a novel Compressed SENSE accelerated ECG- and respiratory-triggered flow-independent 3D isotropic Relaxation-Enhanced Angiography without Contrast and Triggering (modified REACT) with standard non-ECG-triggered 3D contrast-enhanced magnetic resonance angiography (CE-MRA) for imaging of the thoracic aorta in patients with connective tissue diseases (CTD) or other aortic diseases using manual and semiautomatic measurement approaches. This retrospective, single-center analysis of 30 patients (June–December 2018) was conducted by two radiologists, who independently measured aortic diameters on modified REACT and CE-MRA using manual (Multiplanar-Reconstruction) and semiautomatic (Advanced Vessel Analysis) measurement tools on seven levels (inner edge): Aortic annulus and sinus, sinotubular junction, mid- and high-ascending aorta, aortic isthmus, and descending aorta. Bland–Altman analysis was conducted to evaluate differences between the mean values of aortic width and ICCs were calculated to assess interobserver agreement. For each level, image quality was evaluated on a four-point scale in consensus with Wilcoxon matched-pair test used to evaluate for differences between both MRA techniques. Additionally, evaluation time for each measurement technique was noted, which was compared applying one-way ANOVA. When comparing both imaging and measurement methods, CE-MRA (mean difference 0.24 ± 0.27 mm) and the AVA-tool (− 0.21 ± 0.15 mm) yielded higher differences compared to modified REACT (− 0.11 ± 0.11 mm) and the MPR-tool (0.07 ± 0.21 mm) for all measurement levels combined without yielding clinical significance. There was an excellent interobserver agreement between modified REACT and CE-MRA using both tools of measurement (ICC > 0.9). Modified REACT (average acquisition time 06:34 ± 01:36 min) provided better image quality from aortic annulus to mid-ascending aorta (p < 0.05), whereas at distal measurement levels, no significant differences were noted. Regarding time requirement, no statistical significance was found between both measurement techniques (p = 0.08). As a novel non-CE-MRA technique, modified REACT allows for fast imaging of the thoracic aorta with higher image quality in the proximal aorta than CE-MRA enabling a reliable measurement of vessel dimensions without the need for contrast agent. Thus, it represents a clinically suitable alternative for patients requiring repetitive imaging. Manual and semiautomatic measurement approaches provided comparable results without significant difference in time need.

## Introduction

Connective tissue diseases (CTDs) are not uncommon clinical conditions with e.g. Marfan syndrome yielding a prevalence of 1–2 in 10,000 and are associated with various aortic diseases such as dilatation or dissection [1, 2]. To monitor aortic dimensions in patients with CTDs, current guidelines recommend dual imaging consisting of transthoracic echocardiography (TTE) and computed tomography angiography (CTA) or magnetic resonance angiography (MRA). With both modalities to be conducted at diagnosis, TTE should be performed annually while CTA/MRA is to be executed every 5 years or annually if certain factors that exhibit a higher individual risk factor regarding complications are present [3, 4]. Contrast-enhanced MRA (CE-MRA) represents the standard technique for the thorax but raises concerns regarding nephrogenic systemic fibrosis (NSF) in end-stage renal failure, anaphylactic reactions, a potential decreased image quality due to mistiming of image acquisition, and long term retention of gadolinium [5–9].

Hence, different non-CE-MRA techniques have been developed in the past to evaluate the thoracoabdominal vessels in different diseases of the thorax, e.g. CTD, congenital heart disease, and pulmonary hypertension [10–13]. These techniques include approaches based on turbo spin echo, spoiled gradient echo, black blood MRI, 4D flow MRI, steady-state free precession (SSFP), and balanced SSFP (bSSFP); with bSSFP and SSFP being the most widely used techniques [14–20]. Recently, a novel Relaxation-Enhanced Angiography without Contrast and Triggering (REACT) sequence, a combination of non-volume-selective short tau inversion recovery (STIR) pulse, a T2 preparation (T2 prep) pulse, and dual gradient echo Dixon (mDIXON XD) readout, was introduced. As a flow-independent non-CE-MRA, REACT provides high blood-tissue contrast with robust background suppression over a large field of view (FOV) and enables a 3D isotropic readout without physiological triggering or subtraction [21]. As possible limitation, REACT simultaneously depicts both arteries and veins [21]. However, this can also be regarded as an advantage as demonstrated in a recent study investigating the imaging of the pulmonary vasculature in congenital heart disease [22].

Furthermore, reliable repeated measurements of the thoracic aorta in patients with CTDs are a prerequisite to detect disease progression, e.g. a dilatation of the root larger 5 cm, which requires prophylactic aortic root replacement [23]. Manual measurements may be hampered by user-dependency and tend to be more time-consuming [24, 25]. User-independent semiautomatic forms of measurement using specific software may overcome such problems.

The purpose of this study was twofold: first, to examine whether there are differences between a non-ECG-triggered 3D CE-MRA and a novel Compressed SENSitivity Encoding (SENSE) accelerated navigator- and ECG-triggered 3D non-CE-MRA using a modified REACT sequence regarding measurement of aortic diameters and image quality of the thoracic aorta in patients with CTD and other aortic diseases. Second, to investigate the accuracy, reproducibility and time needed for manual (Multiplanar-Reconstruction) and semiautomatic (Advanced Vessel Analysis) measurement approaches of aortic dimensions applied on both imaging techniques.

## Materials and methods

The local institutional review board approved this retrospective, single-center study (reference number: 19-1184) and waived the requirement for written informed consent from the patient cohort.

### Patient population

We retrospectively reviewed our internal database over a 7-month study period (June–December 2018). Scans were included when patients received a dedicated standard protocol for imaging of the thoracic aorta in clinical routine, including both, modified REACT and CE-MRA. Exclusion criteria were severe susceptibility artifacts impairing at least three measurement levels in both CE-MRA and modified REACT as well as insufficient contrast in CE-MRA.

The following data were obtained from the medical charts or observed during examination: Patient age, gender, aortic disease, prior treatment for aortic disease, body mass index (BMI), heart rate, and scan time for MRA sequences.

### Image acquisition

All scans were performed on a clinical whole body 1.5 T MRI scanner (Philips Ingenia; *Philips Healthcare, Best, The Netherlands*) equipped with a dedicated 28-channel coil for cardiac imaging. The protocol comprised modified REACT, CE-MRA and 2D bSSFP breath-hold cine sequences in standard orientations (4-chamber, 2-chamber, 3-chamber, short axis, and aortic sinus).

For non-CE-MRA, imaging was based on a modified flow-independent 3D isotropic REACT sequence, which enables the simultaneous depiction of arteries and veins. REACT exploits the specific relaxation properties of blood and is therefore independent of blood flow [21]. A 50 ms T2 prep sequence was combined with two-point modified DIXON (mDIXON XD; *Philips Healthcare, Best, The Netherlands*) readout. Since the perivascular structures surrounding the thoracic aorta are less pronounced compared to other vascular territories, e.g. the extracranial arteries, background suppression of mDIXON XD in combination with T2 prep was sufficient for cardiovascular applications. Hence, no STIR preparation was employed in the current work, contrary to the original REACT sequence as presented by Yoneyama et al. [21]. To compensate for cardiac and respiratory motion, ECG-triggering (end-diastolic) and respiratory navigator-triggering (diaphragmatic pencil-beam navigation, placed on the right hemidiaphragm, 6 mm gating window during end-expiration) was applied. Therefore and due to the omission of the STIR prepulse, we refer to this sequence as “modified” REACT throughout the manuscript.

For acceleration of image acquisition, Compressed SENSE (*Philips Healthcare, Best, The Netherlands*), a combination of compressed sensing and parallel imaging using SENSE, was used [26, 27]. The method is based on a variable density incoherent sampling pattern for data acquisition, with high-density sampling in the center and continuously decreased sampling density towards the k-space periphery in combination with an iterative L1 norm minimization, assuring data consistency and image sparsity in the wavelet domain for image reconstruction. The reconstruction was regularized by coil sensitivity distribution and SENSE parallel imaging. Images were reconstructed online on the standard hardware as provided by the manufacturer of the MRI system. An acceleration factor of 10 was used, resulting in a nominal scan time of 01:20 min.

Remaining scanning parameters were as following: slice orientation: sagittal; acquisition type: 3D Cartesian; acquired resolution: 1.5 × 1.5 × 1.5 mm^3^; reconstructed resolution: 0.7 × 0.7 × 0.7 mm^3^; field of view (FOV): 400 × 400 × 90 mm^3^; acquisition matrix size: 200 × 203 × 120; k-space lines per heartbeat: 35; flip angle: 15°; TR/TE_1_/TE_2_: 6.0/1.69/3.8 ms.

For CE-MRA, radiofrequency-spoiled T1-weighted gradient echo sequence was used. Gadobutrol (Gadovist; *Bayer HealthCare Pharmaceuticals, Berlin, Germany*; 0.1 ml/kg body weight) was applicated automatically at a flow-rate of 1.5 ml/s (5 ml) and afterwards at a flow-rate of 0.8 ml/s (7 ml) followed by a 20 ml saline flush at a flow-rate of 1.5 ml/s into an antecubital vein. While determining the optimal acquisition time point by a bolus-tracking sequence, patients were asked to perform an end-expiratory breath-hold during acquisition. No triggering was applied. Images were created by subtraction of a native and a CE acquisition. For acceleration, SENSE (*Philips Healthcare, Best, The Netherlands*) with an acceleration factor of 4 was used, resulting in a nominal scan time of 00:25 min.

Remaining scanning parameters were as following: slice orientation: coronal; acquisition type: 3D Cartesian; acquired resolution: 1.5 × 1.5 × 3.6 mm^3^; reconstructed resolution: 0.7 × 0.7 × 1.8 mm^3^; FOV: 450 × 396 × 157 mm^3^; acquisition matrix size: 376 × 286 × 87; flip angle: 35°; TR/TE: 3.4/1.12 ms.

### Measurement

**Fig. 1:**
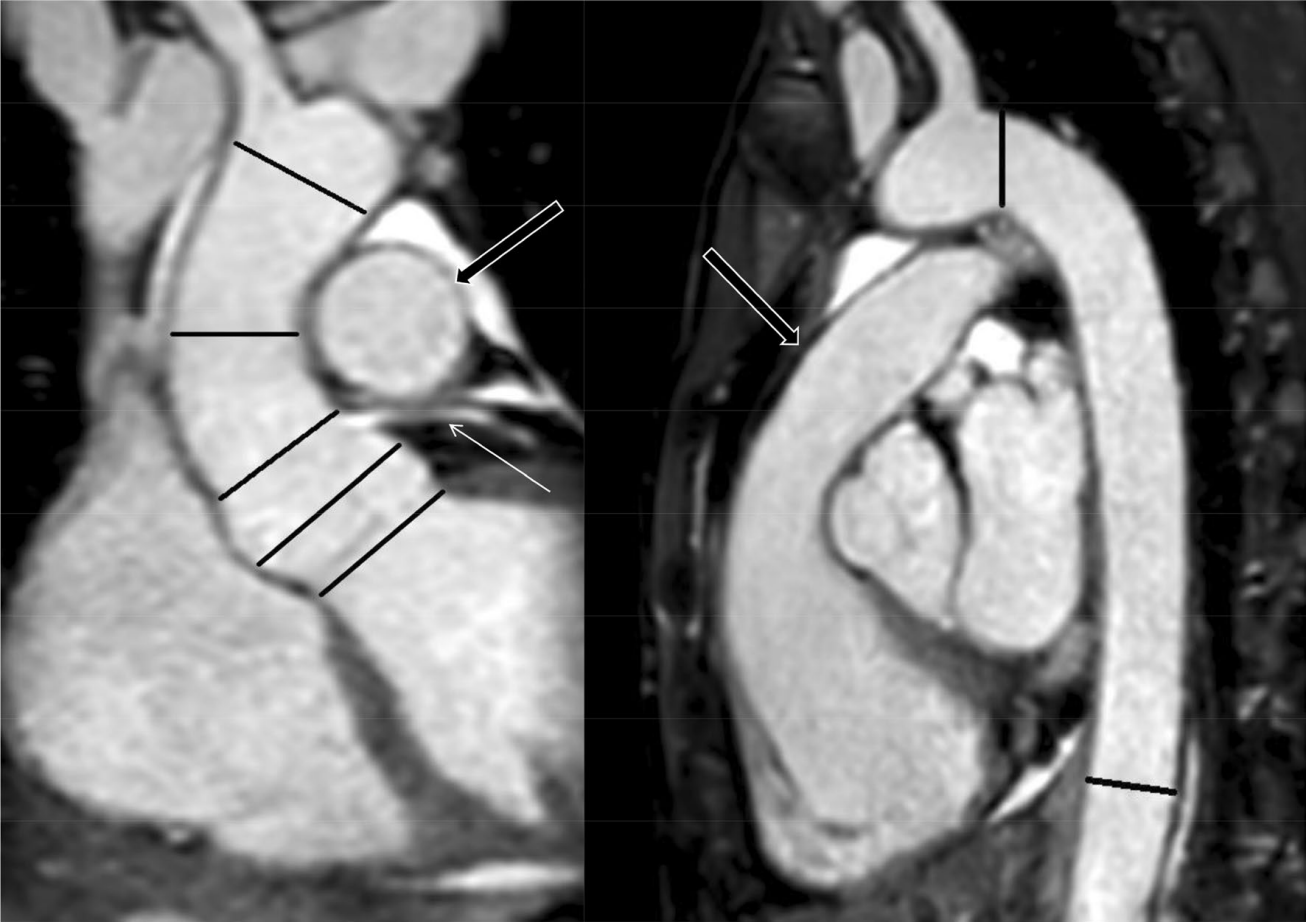
Coronal and sagittal planes of modified REACT (source images) with lines depicting the measurement levels in a 41-year-old female patient with Ehlers-Danlos syndrome. Proximal to distal: Aortic annulus, aortic sinus, sinotubular junction (indicated by the left coronary artery branch; thin arrow), mid- and high-ascending aorta, aortic isthmus, and descending aorta. Note: The main pulmonary artery (wide arrows) is displayed in high quality

Anonymized datasets of REACT and CE-MRA were presented in random order to two radiologists with 2 and 3 years of experience in cardiovascular imaging and MRI. Based on current guidelines of the American Heart Association, both readers independently conducted measurements of aortic diameters on seven distinct levels on source images during separate reading sessions using an inner diameter approach (Fig. 1) [28]:

1. Aortic annulus
2. Aortic sinus (cusp-to-cusp)
3. Sinotubular junction
4. Mid-ascending aorta (2 cm distal of 3.)
5. High-ascending aorta (at the level of the brachiocephalic trunk)
6. Aortic isthmus
7. Descending aorta (at the level of diaphragm passage)

For each level, measurement was conducted using two different approaches: First, the Multiplanar-Reconstruction-(MPR) tool in a commercially available image viewer (*IMPAX EE*; *Agfa HealthCare N.V*., *Mortsel, Belgium*) with manual perpendicular alignment. Second, a semiautomatic approach using the Advanced Vessel Analysis-(AVA) tool of IntelliSpace Portal (V9; *Philips Healthcare, Best, The Netherlands*), which involves manual definition of a centerline along the vascular structure. This results in automated delineation of the vessel wall further allowing orthogonal views of the vessel in cross-section as well as the automated detection of largest and smallest diameters. Regarding the AVA-tool, automatic annulus and sinus measurements were excluded due to known recurring measurement errors in both modalities. In terms of failed automatic vessel delineation at other levels, measurements were adjusted manually; however, modifications were kept minimal. If the automatic vessel delineation performed adequately, no changes were made.

**Fig. 2:**
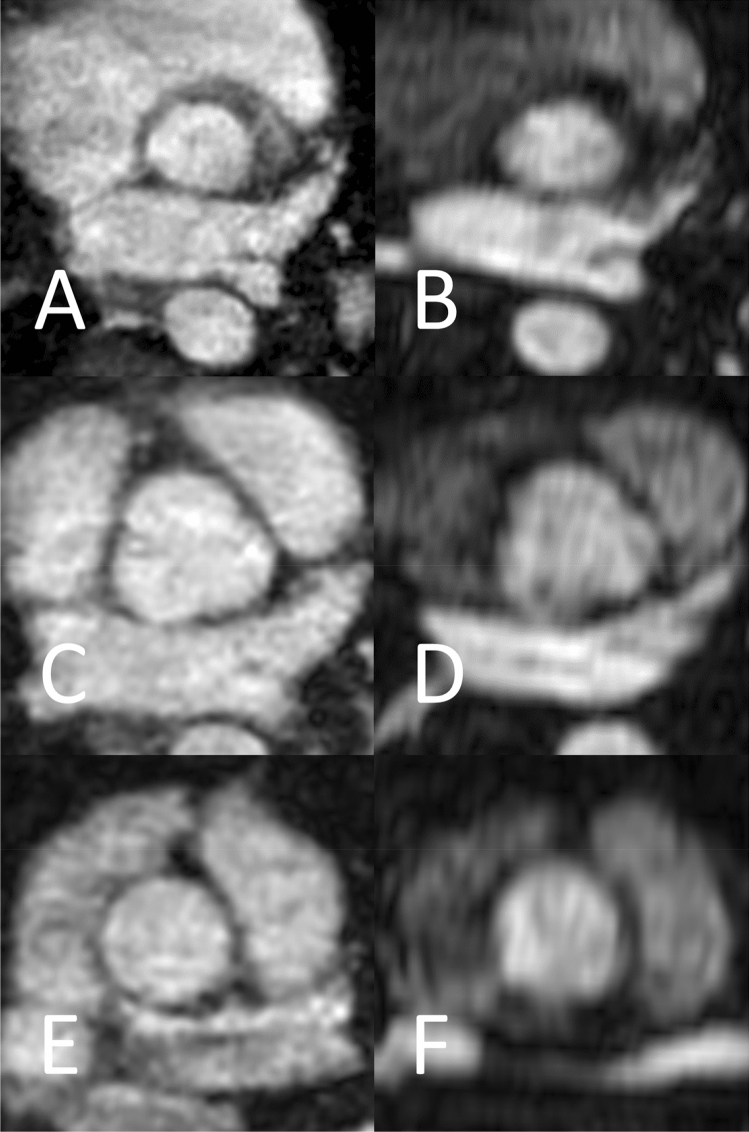
Examples for the levels of measurements in a 34-year-old male patient with Marfan syndrome (same patient as in Fig. 5) at the level of the aortic annulus (**a** and **b**), aortic sinus (**c** and **d**), and sinotubular junction (**e** and **f**) in modified REACT (**a**, **c**, and **e**) and CE-MRA (**b**, **d**, and **f**) in axial plane

Figure 2 shows representative levels of measurement of REACT and CE-MRA in axial plane at the aortic annulus, aortic sinus, and sinotubular junction.

Further, time in minutes was noted for every procedure of measurement using the AVA- and MPR-tool. In general, measurement levels were excluded when they could not be assessed due to severe pulsation or susceptibility artifacts.

### Image quality evaluation

Image quality was evaluated by both observers in consensus regarding anatomic delineation and pulsation artifacts at the levels of measurement using the MPR-tool in all three directions of space to ensure an evaluation uninfluenced by slice orientation of data acquisition. Quality was rated on a Likert scale of 1 to 4: 1 non-diagnostic, 2 poor image quality with severe blurring impairing diagnostic confidence, 3 intermediate image quality with mild blurring, and 4 good image quality without any blurring.

### Statistical analysis

Statistical analysis and graph creation were conducted using Prism (release 8.0.1; *GraphPad Software Inc., San Diego, CA, USA)* and JMP (release 14.1.0; *SAS Institute, Cary, NC, USA*). Data are shown as mean ± standard deviation (SD), unless noted otherwise. Statistical significance was set at p < 0.05. For each level of measurement, the average of all tangential measurements was used for statistical analysis (two measurements at levels 1 and 3–7, three measurements at level 2). Bland–Altman analysis was conducted to evaluate differences between the means regarding the measurement of the aortic width. Wilcoxon matched-pair test was used to evaluate differences regarding image quality. Regarding time need for the different measurement techniques, a one-way ANOVA test was performed. To assess interobserver agreement between measurements obtained from CE-MRA and modified REACT, intraclass correlation coefficients (ICC) were calculated, which were interpreted as follows: < 0.40, poor; 0.40–0.59, fair; 0.60–0.75, good; and 0.75–1.0, excellent agreement [29].

## Results

### Study population and baseline characteristics

**Fig. 3:**
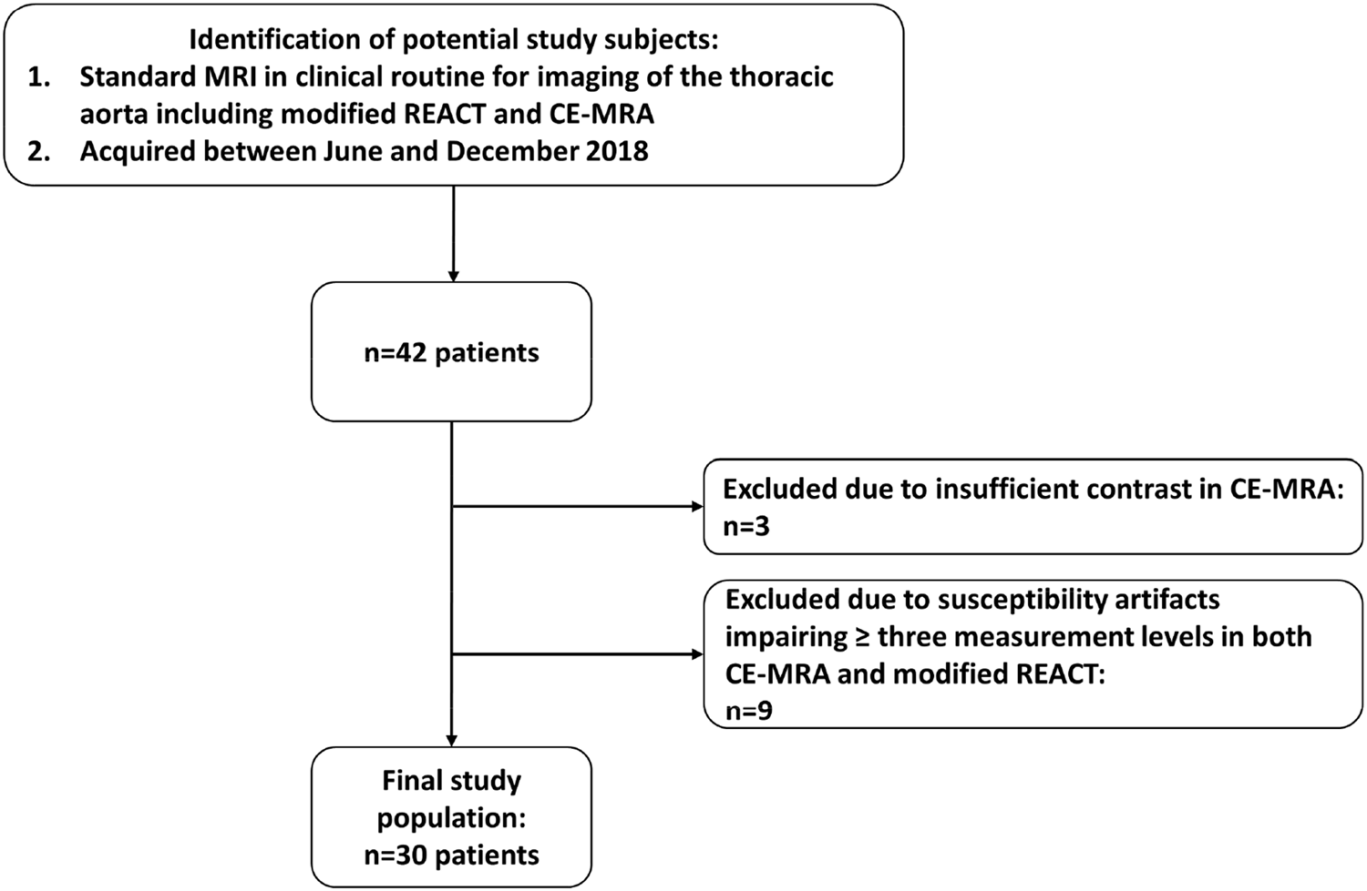
Workflow for inclusion and exclusion of patients

Forty-two patients could be identified. Thereof, three had to be excluded due to insufficient contrast in CE-MRA and nine due to severe susceptibility artifacts in both sequences, resulting in a study population of 30 patients (mean age 38.3 ± 9.5 years, mean body mass index 24.3 ± 3.7, 16 females, average heart rate per minute during examination: 62.4 ± 8.9). A detailed workflow for inclusion and exclusion of study subjects is provided in Fig. 3. 25 patients were clinically verified with genetically determined aortic syndromes due to CTD, the majority being patients with Marfan syndrome (n = 13), approved according to the latest Ghent nosology [2]. Detailed information regarding aortic disease and surgery prior to examination of included patients is given in Table 1.

**Table 1:** Aortic diseases of included patients and treatment received prior to examination

	Number of patients
Aortic disease	
Connective tissue disease	25
Marfan syndrome	13
Ehlers-Danlos syndrome	5
Loeys-Dietz syndrome	4
Familial TAAD	3
Bicuspid aortic valve	2
Coarctation of the aorta	2
Traumatic aortic dissection	1
Surgery prior to examination	
Insertion of aortic prosthesis	5
Valve-sparing aortic root replacement (David procedure)	3
Repair of coarctation of the aorta (CoA)	1

*TAAD* thoracic aortic aneurysm and dissection

### Imaging

All included imaging studies were executed successfully. Modified REACT showed an average total acquisition time of 06:34 ± 01:36 min (including time needed for reconstruction (~ 45 s); depending on the patient’s heart rate and breathing frequency, navigator efficiency: ~ 24%), CE-MRA of 03:09 ± 00:34 min (including time needed for bolus-tracking sequence, reconstruction, and subtraction of the pre-contrast mask).

### Comparison between modified REACT/CE-MRA and between MPR-/AVA-tool

Table 2 provides the average measurement diameters of the thoracic aorta of CE-MRA and modified REACT applying both measurement tools. When comparing both tools of measurement, modified REACT yielded lower differences than CE-MRA (− 0.11 ± 0.11 vs. 0.24 ± 0.27 mm) for all measurement levels combined. When comparing both methods of imaging, the MPR-tool showed lower differences compared to the AVA-tool (0.07 ± 0.21 vs. − 0.21 ± 0.15 mm) considering the combination of all measurement levels.

**Table 2:** Mean absolute and relative differences between both methods of imaging and measurement for all measurement levels combined

	CE-MRA vs. modified REACT (MPR)	CE-MRA vs. modified REACT (AVA)	CE-MRA (MPR vs. AVA)	Modified REACT (MPR vs. AVA)
Differences, mean, mm, SD	0.07 ± 0.21	− 0.21 ± 0.15	0.24 ± 0.27	− 0.11 ± 0.11
95% confidence interval, mm	− 0.35 to 0.48	− 0.50 to 0.08	− 0.28 to 0.76	− 0.32 to 0.12
Differences, relative, %, SD	0.35 ± 0.92	− 0.75 ± 0.44	0.96 ± 0.96	− 0.42 ± 0.41
95% confidence interval, %	− 1.46 to 2.16	− 1.62 to 0.12	− 0.93 to 2.84	− 1.22 to 0.38

*Modified REACT* modified relaxation-enhanced angiography without contrast and triggering, *CE-MRA* contrast-enhanced magnetic resonance angiography, *MPR* Multiplanar-Reconstruction, *AVA* advanced vessel analysis, *SD* standard deviation

**Fig. 4:**
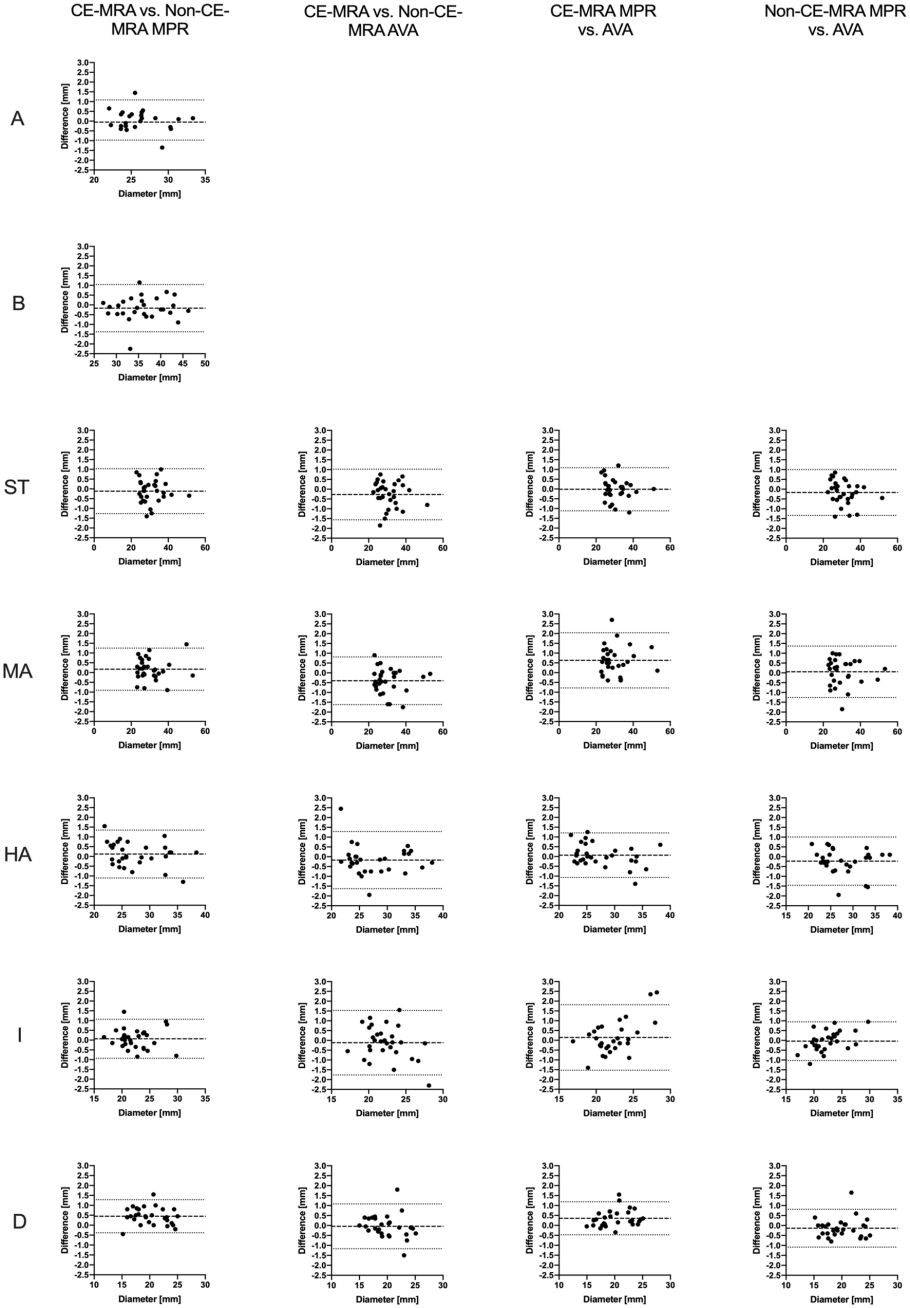
Bland–Altman comparison of measured diameters of the thoracic aorta at the seven levels of measurement (*A* aortic annulus, *B* aortic sinus, *ST* sinotubular junction, *MA* mid-ascending aorta, *HA* high-ascending aorta, *I* aortic isthmus, *D* descending aorta) for the different methods of imaging and measurement. The middle lines represent the mean absolute difference of measurements, the outer boundaries the 95% confidence interval. Due to known recurrent measurement errors of the AVA-tool, measurements at aortic annulus and sinus were excluded

Regarding the comparison of each level individually, the majority yielded differences lower than 0.5 mm for both methods of imaging and measurement, respectively. The highest differences were observed for the descending aorta (0.45 mm/2.31% when comparing CE-MRA and modified REACT using the MPR-tool) and the mid-ascending aorta (0.63 mm/2.15% when comparing the AVA- and MPR-tool in CE-MRA). Detailed results are presented in Tables 3 and 4 as well as Fig. 4.

**Table 3:** Average diameters for both methods of imaging and for both methods of measurement at every level

	Aortic annulus	Aortic sinus	Sinotubular junction	Mid-ascending aorta	High-ascending aorta	Aortic isthmus	Descending aorta
Modified REACT (MPR), mean, diameter, mm, SD	26.23 ± 3.16	36.11 ± 4.97	30.93 ± 6.29	30.38 ± 7.47	27.53 ± 4.66	22.3 ± 2.97	19.92 ± 3.04
95% confidence interval	25.03 to 27.43	34.25 to 37.97	28.58 to 33.28	27.59 to 33.17	25.79 to 29.27	21.19 to 23.41	18.76 to 21.07
Modified REACT (AVA), mean, diameter, mm, SD	–	–	31.1 ± 6.45	30.33 ± 7.52	27.76 ± 4.76	22.34 ± 2.71	20.06 ± 3.00
95% confidence interval	–	–	28.69 to 33.51	27.52 to 33.13	25.98 to 29.54	21.33 to 23.35	18.91 to 21.2
CE-MRA (MPR), mean, diameter, mm, SD	26.18 ± 2.9	35.94 ± 5.19	30.81 ± 6.27	30.55 ± 7.5	27.65 ± 4.48	22.36 ± 2.94	20.37 ± 2.98
95% confidence interval	24.95 to 27.4	33.93 to 37.95	28.47 to 33.15	27.75 to 33.35	25.98 to 29.32	21.27 to 23.46	19.23 to 21.5
CE-MRA (AVA), mean, diameter, mm, SD	–	–	30.82 ± 6.37	29.9 ± 7.45	27.58 ± 4.68	22.22 ± 2.45	20.01 ± 2.88
95% confidence interval	–	–	28.44 to 33.2	27.11 to 32.7	25.83 to 29.33	21.31 to 23.13	18.92 to 21.11

Due to known recurrent measurement errors of the AVA-tool, measurements at aortic annulus and sinus were excluded

*Modified REACT* modified relaxation-enhanced angiography without contrast and triggering, *CE-MRA* contrast-enhanced magnetic resonance angiography, *MPR* Multiplanar-Reconstruction, *AVA* advanced vessel analysis, *SD* standard deviation

**Table 4:** Mean relative differences between both methods of imaging and for both methods of measurement at every level

	CE-MRA vs. modified REACT (MPR)	CE-MRA vs. modified REACT (AVA)	CE-MRA (MPR vs. AVA)	Modified REACT (MPR vs. AVA)
Aortic annulus	0.26 ± 2.01 − 3.68 to 4.19	–	–	–
Aortic sinus	− 0.50 ± 1.77 − 3.97 to 2.98	–	–	–
Sinotubular junction	− 0.38 ± 2.03 − 4.35 to 3.60	− 0.86 ± 2.24 − 5.25 to 3.53	0.02 ± 2.00 − 3.84 to 3.88	− 0.46 ± 2.03 − 4.44 to 3.51
Mid-ascending aorta	0.58 ± 1.83 − 3.01 to 4.17	− 1.35 ± 2.14 − 5.54 to 2.83	2.15 ± 2.50 − 2.75 to 7.05	0.22 ± 2.37 − 4.42 to 4.85
High-ascending aorta	0.55 ± 2.38 − 4.11 to 5.21	− 0.57 ± 3.05 − 6.54 to 5.40	0.36 ± 2.14 − 3.83 to 4.55	− 0.76 ± 2.26 − 5.19 to 3.66
Aortic isthmus	0.32 ± 2.23 − 4.05 to 4.69	− 0.43 ± 3.65 − 7.58 to 6.72	0.42 ± 3.52 − 6.47 to 7.32	− 0.33 ± 2.32 − 4.87 to 4.22
Descending aorta	2.31 ± 2.25 − 2.10 to 6.72	− 0.14 ± 2.70 − 5.42 to 5.15	1.72 ± 2.05 − 2.31 to 5.74	− 0.73 ± 2.37 − 5.38 to 3.92

Values are given in % ± standard deviation with corresponding 95% confidence interval. Due to known recurrent measurement errors of the AVA-tool, measurements at aortic annulus and sinus were excluded

*Modified REACT* modified relaxation-enhanced angiography without contrast and triggering, *CE-MRA* contrast-enhanced magnetic resonance angiography, *MPR* multiplanar-reconstruction, *AVA* advanced vessel analysis, *SD* standard deviation

### Interobserver agreement

For all levels of measurement combined, there was an excellent (ICC > 0.9) interobserver agreement for both methods of imaging and measurement, respectively (Table 5). However, CE-MRA using the MPR-tool yielded a slightly lower interobserver agreement than modified REACT using both methods of measurement and CE-MRA using the AVA-tool.

**Table 5:** Interobserver correlation between both readers for both methods of imaging and measurement for all levels combined

	Modified REACT (MPR)	Modified REACT (AVA)	CE-MRA (MPR)	CE-MRA (AVA)
Differences, mean, mm, SD	− 0.06 ± 0.19	− 0.05 ± 0.18	0.14 ± 0.16	0.13 ± 0.24
95% Confidence interval, mm	− 0.24 to 0.11	− 0.27 to 0.17	− 0.01 to 0.29	− 0.17 to 0.42
Intraclass correlation coefficient	0.994	0.993	0.986	0.993

*Modified REACT* modified relaxation-enhanced angiography without contrast and triggering, *CE-MRA* contrast-enhanced magnetic resonance angiography, *MPR* Multiplanar-Reconstruction, *AVA* advanced vessel analysis, *SD* standard deviation

### Comparison of image quality between modified REACT and CE-MRA

Modified REACT provided a sharper delineation of the aortic root and the proximal ascending aorta resulting in significantly higher image quality scores (p < 0.05). Due to impaired image quality in CE-MRA at the aortic root, measurements could not be performed at the aortic annulus in six and at the aortic sinus in three patients. At distal levels, no significant differences were noted (p > 0.05). In modified REACT, no measurements had to be excluded due to poor image quality. Results of image quality evaluation are provided in Table 6.

**Table 6:** Mean subjective image scores at the seven levels of measurement defined by consensus reading using a four-point Likert scale

	Aortic annulus	Aortic sinus	Sinotubular junction	Mid-ascending	High-ascending	Aortic isthmus	Descending aorta
Modified REACT	3.69	3.7	3.73	3.8	3.97	3.8	3.9
CE-MRA	2.48	2.5	3.1	3.67	3.9	3.73	3.86
P value	** < 0.0001**	** < 0.0001**	** < 0.0001**	**0.02**	0.17	0.35	0.7

For comparison of differences, Wilcoxon matched-pair analysis was used, bold indicating significant differences

*Modified REACT* modified relaxation-enhanced angiography without contrast and triggering, *CE-MRA* contrast-enhanced magnetic resonance angiography

**Fig. 5:**
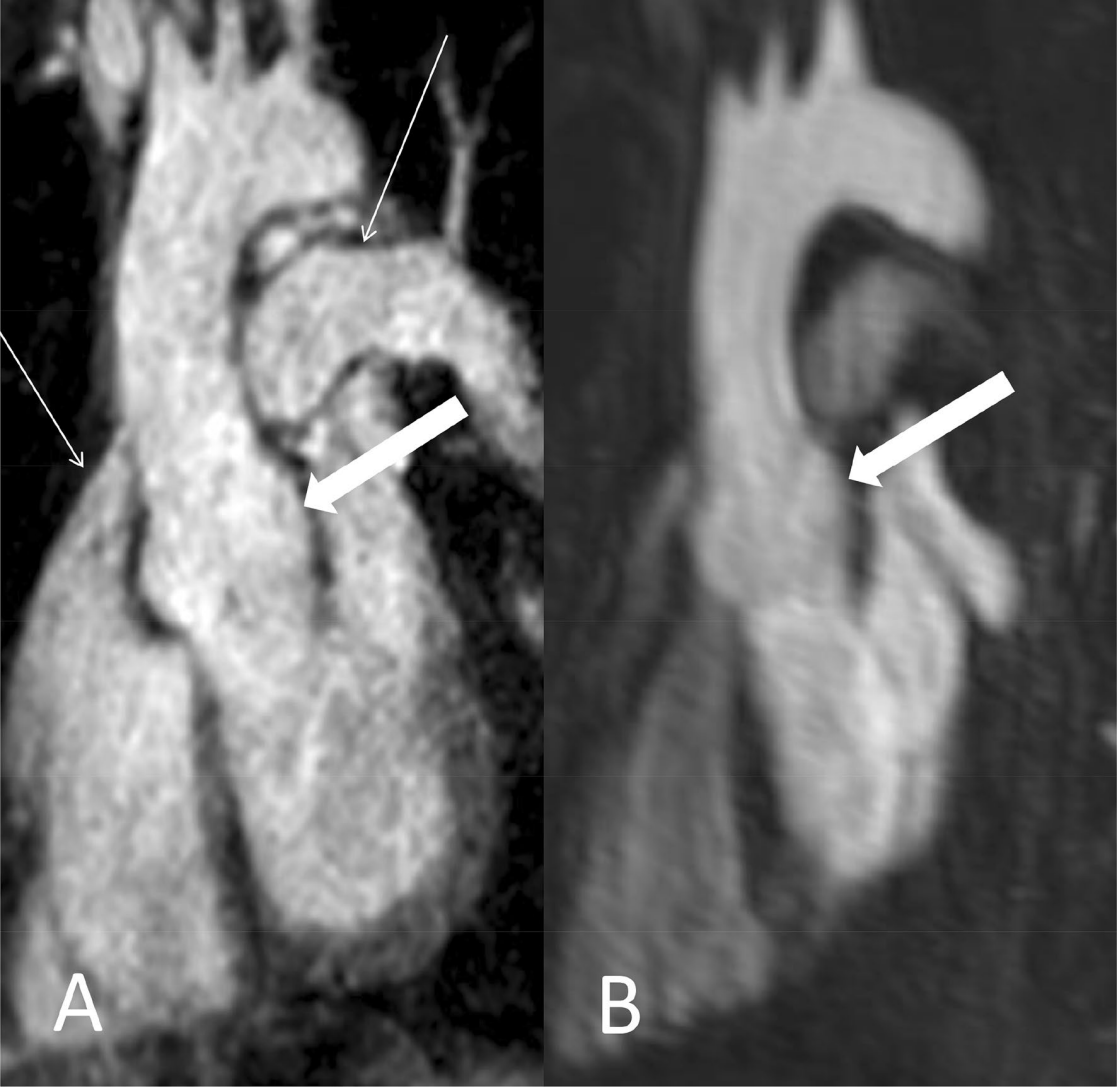
Parasagittal planes (source images) of modified REACT (**a**) and CE-MRA (**b**) in a 34-year-old male patient with Marfan syndrome. While image quality is comparable at the aortic arch, modified REACT provides better delineation of the vessel wall at the aortic root than CE-MRA given pulsation artifacts of the latter. Additionally, modified REACT enables the depiction of the pulmonary arteries in high quality (thin arrows)

**Fig. 6:**
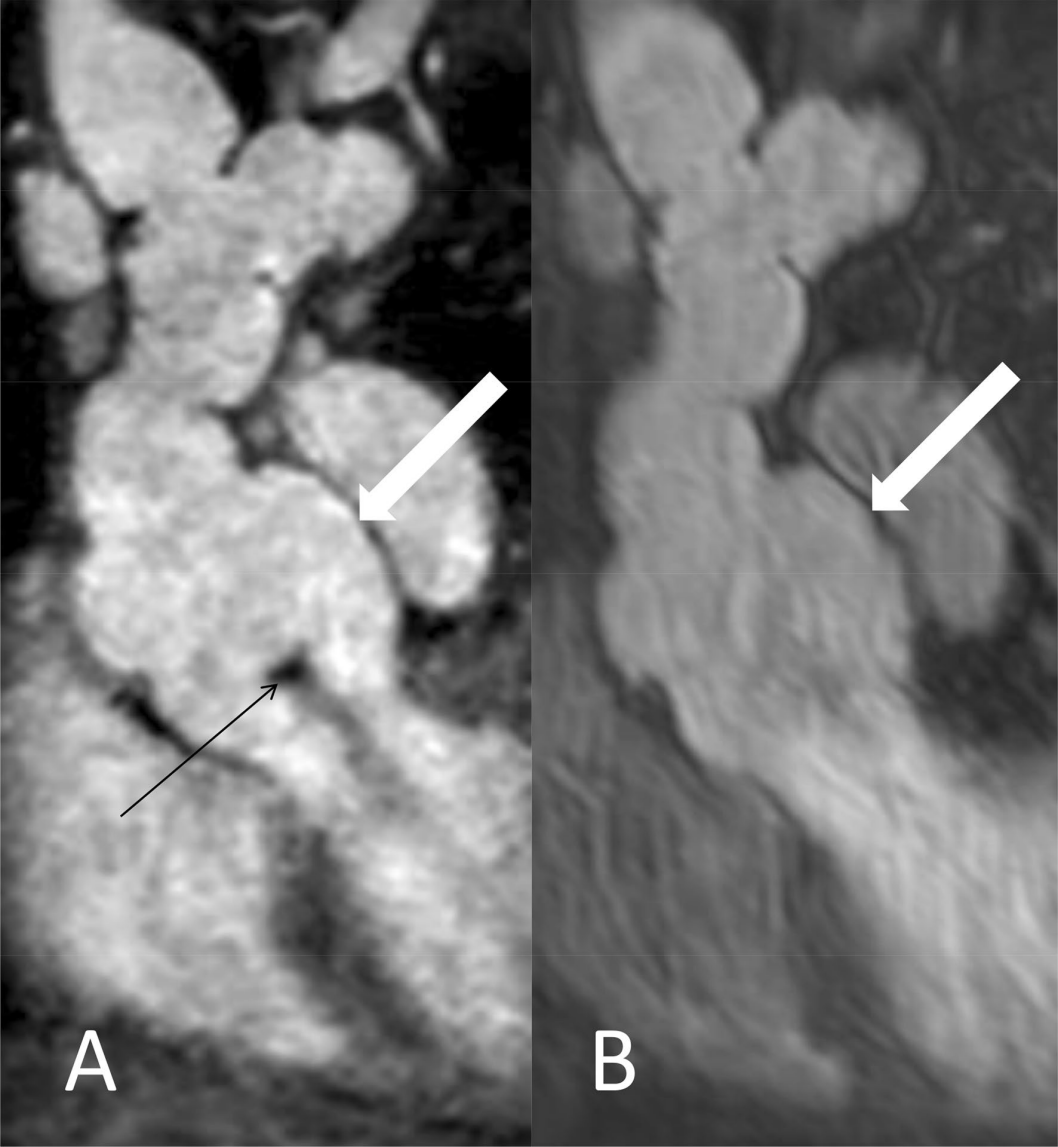
Coronal planes (source images) of modified REACT (**a**) and CE-MRA (**b**) in a 46-year-old male patient with Marfan syndrome ten years after valve-sparing aortic root replacement (David procedure) and replacement of the proximal aortic arch. Modified REACT enables an improved delineation of the thoracic aorta, pronounced at the aortic root (wide arrows) due to light mistiming of image acquisition regarding first pass of the contrast bolus and pulsation artifacts hampering image quality in CE-MRA. Note the clearly visible aortic regurgitation in modified REACT (thin arrow), indicating aortic insufficiency

**Fig. 7:**
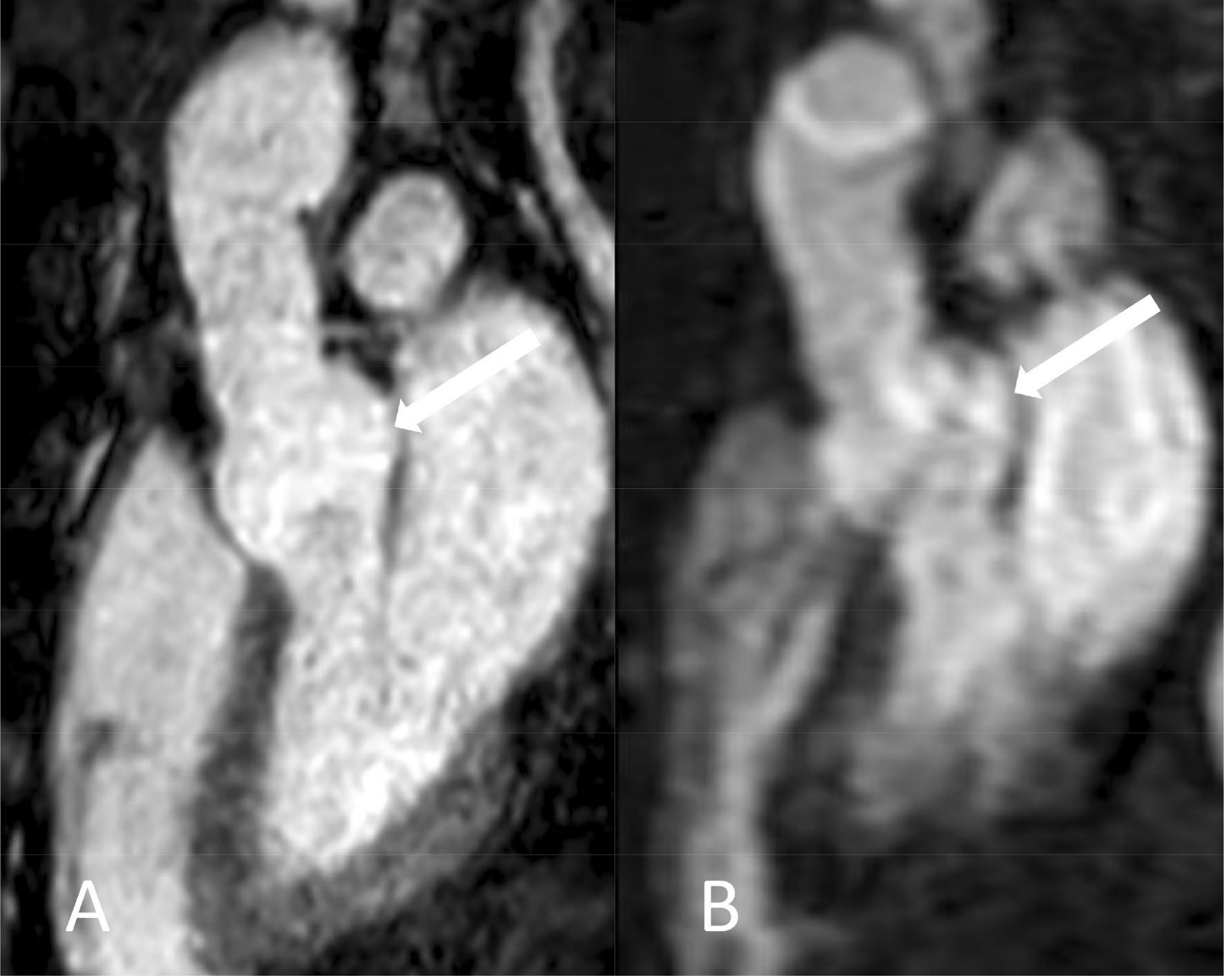
Sagittal planes (source images) of modified REACT (**a**) and CE-MRA (**b**) in a 60-year-old male patient with Marfan syndrome two years after valve-sparing aortic root replacement (David procedure). Modified REACT provides an improved depiction of the thoracic aorta, pronounced at the root (arrows) with pulsation artifacts impeding image quality of CE-MRA

Figures 5, 6 and 7 give exemplary comparisons of modified REACT and CE-MRA. Of note, the pulmonary arteries are displayed in high quality using modified REACT (Figs. 1 and 5).

### Time need for MPR- and AVA-tool

MPR-based measurements showed a lower time need (05:50 ± 01:00 min (modified REACT), 05:40 ± 01:00 min (CE-MRA)) compared to AVA-tool based measurements (06:14 ± 1:08 min (modified REACT), 05:46 ± 01:00 min (CE-MRA)) (p = 0.08).

## Discussion

In this study, we compared a novel Compressed SENSE accelerated navigator- and ECG-triggered flow-independent 3D isotropic modified REACT with a non-ECG-triggered 3D CE-MRA for imaging of the thoracic aorta in patients with CTD or other aortic diseases using manual (MPR) and semi-automatic (AVA) measurement approaches. The major findings of this study were the following: Measurement differences between both methods of imaging and measurement at every aortic level individually were small with the majority yielding differences lower than 0.5 mm. For all measurement levels combined, there were lower differences for modified REACT when comparing both measurement methods and for the MPR-tool when comparing both imaging techniques. An excellent interobserver agreement was observed for both methods of imaging and measurement. Modified REACT scored significantly higher image quality values at the proximal aorta, and the application of the MPR-tool was less time consuming without yielding a statistical significance.

In line with previous studies, which compared other ECG- and respiratory triggered non-CE-MRA techniques such as 3D SSFP with CE-MRA for imaging of the thoracic aorta, there were only small differences regarding measurement between both methods of imaging obtained by both measurement approaches [20, 30]. For all measurement levels combined, highest differences were noted in CE-MRA when comparing both tools of measurement (0.24 mm), mostly due to pulsation and breathing artifacts. Considering the levels individually, the descending aorta (0.45 mm, when comparing CE-MRA and modified REACT using the MPR-tool) and the mid-ascending aorta (0.63 mm, when comparing the AVA- and MPR-tool in CE-MRA) showed the highest differences, most likely due to a different height of measurement. However, these differences were of no clinical significance since current guidelines state that only discrepancies indicative of a threshold growth of > 5 mm over time are to be considered relevant [4]. Further, the majority of levels showed differences smaller than 0.5 mm, in line with previous studies [20, 30]. Modified REACT showed lower differences between both readers than CE-MRA using both measurement tools. However, both methods of imaging showed high ICCs as similar studies stated, also given the fact that for nine patients, proximal levels of measurement were excluded in CE-MRA given severe pulsation and breathing artifacts with hampered vessel delineation [20, 30, 31]. Their inclusion would have potentially led to decreased ICCs for CE-MRA due to impaired assessment of vessel boundaries.

Aforementioned and other studies have already demonstrated that ECG-triggering and respiratory-gating of other non-CE-MRA techniques such as SSFP lead to suppression of pulsation and breathing artifacts, consequently outperforming untriggered CE-MRA in terms of image quality, which was considered as the standard of reference in these studies [11, 16, 20, 31–33]. In the current study, we could confirm these findings as modified REACT with respective triggering provided significant higher image quality scores at the proximal aorta than CE-MRA, consequently facilitating the assessment of the aortic root (Figs. 5, 6 and 7).

The MPR-tool, although being a manual device and despite discarding measurements at aortic annulus and sinus when using the AVA-tool, resulted in lower measurement time than the AVA-tool without reaching statistical significance. The findings of this study are opposed to a CTA-study in which a different semiautomatic tool was less time consuming, potentially due to a different method of semiautomatic measurement (MeVisLab vs. IntelliSpace Portal) with difference in time needed for data processing [24]. Further, the AVA-tool gave rise to higher measurement differences than the MPR-tool when comparing its use in modified REACT and CE-MRA. Moreover, there were recurrent errors at the location of the aortic annulus and sinus in both modified REACT and CE-MRA using the AVA-tool, requiring exclusion of these levels. To our knowledge, there are no studies investigating the measurement at these locations in MRA by means of a semiautomatic tool, most likely due to these errors. Nevertheless, it needs to be considered that the usage of the MPR-tool requires a certain expertise to repeatedly achieve an accurate perpendicular alignment at each location. Further, the AVA-tool allows illustration of the locations of measurement along the entire course of the vessel, which then can be saved to a single image. Moreover, it produces automated features such as identification of the maximum vessel width in a given segment allowing monitoring of the maximal width over time, therefore facilitating repeated measurements in follow-up studies, especially for unexperienced readers [24, 34].

The recently introduced 3D flow-independent isotropic REACT technique overcomes known limitations of 3D SSFP/bSSFP imaging, such as sensitivity to off-resonance effects, disruptions of the steady state due to highly pulsatile flow, and long acquisition times to cover large FOVs, by the following: First, given its mDIXON XD technique for water/fat separation, REACT is widely insensitive to inhomogeneities in the magnetic field, making it suitable for the application at higher magnetic fields such as 3 T as well, where inhomogeneities are expected to be higher [21, 35]. Second, with the application of Compressed SENSE, modified REACT yielded reconstruction times below one minute and an overall scan time of 06:34 min, lower than 3D SSFP (up to 10 min) for the same kind of investigation and FOV [11, 27, 30, 33, 36]. Of note, the time needed for image reconstruction was not explicitly reported in these studies; hence, the total acquisition time can be expected to be longer. To compensate for respiratory and cardiac motion, REACT was combined with respective triggering in this study. In principle, REACT, given its flow-independency, may be used without ECG- or respiratory-triggering making it a versatile alternative to CE-MRA, especially for patients who are unable to perform a breath-hold, e.g. children. As recently shown, modified REACT enables the simultaneous display of the different vessels of the pulmonary vasculature in high quality with visually comparable signal intensity, which can be differentiated sufficiently given less pronounced perivascular structures and their distinct location [22]. Given the results of the present study, modified REACT therefore offers the possibility of evaluating the vascular territories of the thorax using one single acquisition (Figs. 1 and 5). Nevertheless, it needs to be considered that the CE-MRA used in this study allows the imaging of the thoracoabdominal aorta in one single acquisition, whereas modified REACT provides a smaller FOV only displaying the thoracic aorta and the superior part of the abdominal aorta.

### Limitations

Besides its retrospective, single-center setting, our study has some limitations. First, given the obvious differences in appearance, readers were not blinded to the type of sequence potentially influencing the results. Second, the usage of individual MPRs and the potential user influence may be seen as a disadvantage of this work [24, 25]. On the other hand, due to the oval configuration of the aorta, recent studies showed that the measured vessel width yields on exact image orientation, which may only be achieved by using perpendicular alignment [16, 33]. Third, no direct comparison to other non-CE-MRA techniques, e.g. 3D SSFP, was conducted in this study, which could nurture future research. Fourth, the lower, anisotropic resolution of CE-MRA consequently leads to an inferior image quality than modified REACT influencing the results of image quality evaluation and the accuracy of diameter measurement. However, the resolution of applied CE-MRA in the current study is similar to recent studies comparing other non-CE-MRA techniques with CE-MRA [16, 30, 31]. Fifth, the comparison of the triggered modified REACT with a non-triggered breath-hold first-pass CE-MRA sequence can be considered as a limitation of this work, especially since it has been shown that ECG-gating improves the image quality of CE-MRA [37]. However, ECG-gated CE-MRA does not provide the same high image quality of ECG- and navigator gated non-CE-MRA techniques such as 3D SSFP for the aortic root [11, 30]. Additionally, ECG-gating proves to be challenging and leads to prolonged scan time potentially resulting in suboptimal contrast phase, especially in bradycardic patients; therefore, non-ECG-gated CE-MRA is still the most widely used technique in clinical routine [38].

## Conclusion

Compressed SENSE accelerated 3D modified REACT allows for robust and reliable imaging of the thoracic aorta with higher image quality at the proximal aorta than CE-MRA without the necessity of contrast agent. Given its short acquisition time, it represents a clinically applicable method for patients requiring repetitive imaging of the thoracic aorta.

## Data Availability

The datasets generated and/or analyzed during the study are not publicly available due to data protection but are available from the corresponding author upon reasonable request.
